# Perioperative brain injury marker concentrations in neonatal open-heart surgery: a prospective observational study

**DOI:** 10.3389/fped.2023.1186061

**Published:** 2023-08-09

**Authors:** Åsa Jungner, Finn Lennartsson, Isabella Björkman-Burtscher, Kaj Blennow, Henrik Zetterberg, David Ley

**Affiliations:** ^1^ Pediatric Surgery and Neonatal Care, Department of Clinical Sciences Lund, Lund University, Skåne University Hospital, Lund, Sweden; ^2^ Diagnostic radiology, Department of Clinical Sciences Lund, Lund University, Skåne University Hospital, Lund, Sweden; ^3^ Department of Radiology, Sahlgrenska University Hospital, Region Västra Götaland, Gothenburg, Sweden; ^4^ Department of Radiology, Institute of Clinical Sciences, Sahlgrenska Academy, University of Gothenburg, Gothenburg, Sweden; ^5^ Department of Psychiatry and Neurochemistry, Institute of Neuroscience and Physiology, Sahlgrenska Academy, University of Gothenburg, Mölndal, Sweden; ^6^ Clinical Neurochemistry Laboratory, Sahlgrenska University Hospital, Region Västra Götaland, Mölndal, Sweden; ^7^ Department of Neurodegenerative Disease, UCL Institute of Neurology, London, UK; ^8^ UK Dementia Research Institute at UCL, London, UK; ^9^ Hong Kong Center for Neurodegenerative Diseases, Clear Water Bay, Hong Kong, Hong Kong SAR, China; ^10^ Wisconsin Alzheimer’s Disease Research Center, University of Wisconsin School of Medicine and Public Health, University of Wisconsin-Madison, Madison, WI, United States

**Keywords:** congenital heart defect, neonatal open-heart surgery, cardiopulmonary bypass, white matter injury, brain injury marker, glial fibrillary acidic protein, neurofilament light, tau

## Abstract

Neonates with critical congenital heart defects undergoing open-heart surgery on cardiopulmonary bypass circulation are at risk for white matter brain injury. This article reports on pre- and postoperative plasma concentrations of brain injury markers glial fibrillary acidic protein (GFAP), neurofilament light (NfL) and Tau, and their respective associations with white matter lesions detected on postoperatively performed brain MRI. Forty term newborns with isolated critical congenital heart defects were included in a prospective observational study. Brain injury marker plasma concentrations were determined prior to surgery and at postoperative days 1, 2 and 3. Brain magnetic resonance imaging was performed pre- and postoperatively. Concentrations of brain injury markers were analysed using ultrasensitive single molecule array technology. Absolute pre- and postoperative plasma biomarker concentrations, and postoperative concentrations adjusted for preoperative concentrations were used for subsequent analysis. Plasma concentrations of GFAP, NfL and Tau displayed a well-defined temporal trajectory after neonatal cardiopulmonary bypass circulation. GFAP and Tau reached peak concentrations at postoperative day 2 (median concentrations 170.5 and 67.2 pg/ml, respectively), whereas NfL continued to increase throughout the study period (median concentration at postoperative day 3 191.5 pg/ml). Adjusted Tau at postoperative day 2 was significantly higher in infants presenting with white matter lesions on postoperative MRI compared to infants without white matter injury.

## 1. Introduction

Neonates with critical congenital heart defects requiring open-heart surgery in infancy are at risk for white matter brain injury and impaired neurodevelopment (1,2). A prognostic biomarker enabling identification of newborns at risk would be beneficial, recognizing the often subtle clinical manifestations of white matter aberrations in infancy and the possible long-term consequences of neonatal brain white matter injury (3,4).

Glial fibrillary acidic protein (GFAP), Neurofilament Light (NfL) and Tau are recognized biomarkers for glial and axonal brain injury. GFAP constitutes a key element in astrocyte structure and function (5). Increased plasma concentrations of GFAP after pediatric cardiopulmonary bypass circulation (CPB) have been associated with nadir oxygen delivery (6) and nadir temperature (7) during bypass circulation. Moreover, GFAP concentrations at bypass separation in a neonatal cohort have been correlated to adverse neurodevelopmental outcomes at 12 months of age (8). NfL is a cytoskeletal protein predominantly found in myelinated axons (9). The potential use of NfL as a biomarker for adverse neurodevelopmental outcome has been evaluated in preterm cohorts (10,11), but never in infants with critical congenital heart defects. Tau is located mainly in axons and has emerged as a promising predictor for brain injury as determined by magnetic resonance imaging (MRI) and adverse neurocognitive outcome in newborns with hypoxic-ischemic encephalopathy (HIE) (12,13). A small-scale clinical trial suggested an association between neurological injury and increased Tau concentrations in children treated with extracorporeal membrane oxygenation (14), but perioperative concentrations during neonatal open-heart surgery have not yet been evaluated.

The primary aim of this study was to evaluate the temporal trajectory of GFAP, NfL and Tau plasma concentrations before and after neonatal open heart surgery. The secondary aims were to evaluate the association between perioperative plasma brain injury marker concentrations and structural postoperative white matter injury, and to examine the potential correlations between selected clinical variables and pre- and postoperative biomarker concentrations.

## 2. Material and methods

### 2.1. Ethical considerations

The study was conducted according to the guidelines of the World Medical Association Declaration of Helsinki and approved by the Swedish Ethical Review Authority (protocol code 2014/479). Study participants were included after written informed consent from the neonates’ legal guardians.

### 2.2. Study cohort and execution

Forty term neonates with isolated critical congenital heart defects requiring surgery on CPB circulation within 30 days of life were included in a prospective observational study. In order to avoid confounding, neonates with suspected or confirmed syndrome diagnosis, hypoxic-ischemic encephalopathy grades 2–3, or with the requirement for preoperative support on extracorporeal membrane oxygenation were excluded.

Blood samples for determination of plasma brain injury marker concentrations were obtained preoperatively at the day of surgery and at postoperative days 1, 2 and 3. Sampling was discontinued when the arterial line was withdrawn. Blood samples were collected in sodium citrate tubes and immediately centrifuged. Plasma was separated, aliquoted into 200 μL portions in cryo tubes and snap-frozen on dry ice. Samples were stored at −80${}^{\circ }$C until analysis.

Preoperative brain magnetic resonance imaging (MRI) was performed in as close proximity to surgery as possible. Postoperative brain MRI was performed in hemodynamically stable neonates following removal of myocardial electrodes. Clinical characteristics of the study participants are reported in Table 1.

**Table 1:** Clinical characteristics of included study participants.

Clinical characteristics of the study cohort	
Sex (male/female) (n)	25/15
Birthweight (g) (median (IQR))	3357 (3234–3592)
Gestational age at birth (weeks) (median (min–max))	39+4 (37+6–42+1)
Preoperative balloon atrial septostomy (n)	13/40
Maximal preoperative lactate (mmol/L) (median (IQR))	2.8 (2.2–4.0)
Preoperative white matter lesion (n)	1/24
Postnatal age at surgery (days) (median (IQR))	5 (4–7)
RACHS–1 (median (range))	4 (3–6)
Corrective procedure	
ASO + VSD closure (n)	11/40
ASO (n)	9/40
repair of hypoplastic arch + VSD closure (n)	5/40
repair of truncus arteriosus (n)	2/40
ASO + repair of hypoplastic arch + VSD closure (n)	1/40
repair of hypoplastic arch (n)	1/40
repair of TAPVR with obstruction (n)	1/40
Yasui procedure (n)	1/40
aortic valvotomy (n)	1/40
Palliative procedure	
Norwood procedure (n)	3/40
BT-shunt + atrial septectomy + pulmonary valvotomy (n)	2/40
BT-shunt + atrial septectomy (n)	2/40
BT-shunt + pulmonary arterioplasty (n)	1/40
Time on CPB (min) (median (IQR))	182 (142–208)
Ultrafiltration (n)	23/40
Selective cerebral perfusion (n)	11/40
Maximal postoperative lactate (mmol/L) (median (IQR))	3.1 (2.3–3.9)
Postoperative ECMO (n)	0/40
Postoperative white matter lesion (n)	16/30

RACHS, risk adjustment for congenital heart surgery, ASO, arterial switch operation; VSD, ventricular septal defect; TAPVR, total anomalous pulmonary venous return; BT-shunt, Blalock-Taussig shunt; CPB, cardiopulmonary bypass; ECMO, extracorporeal membrane oxygenation.

### 2.3. Analysis of plasma brain injury markers

Plasma NfL, GFAP and Tau concentrations were measured by single molecule array (Simoa) technology using the commercially available Neuro 4-plex A kit on an HD-X instrument according to instructions from the kit manufacturer (Quanterix, Billerica, MA). The measurements were performed in one round of experiments, using one batch of reagents by board-certified laboratory technicians who were blinded to clinical data. Intra-assay coefficients of variation, monitored using internal quality control samples (one high and one low sample in duplicates on each plate), varied from 5.3–11%.

### 2.4. Magnetic resonance imaging

Brain MRI was performed on a 3T MR scanner (PRISMA, Siemens Healtheneers, Erlangen, Germany) using a 20 channel receive head coil (Siemens Healtheneers, Erlangen, Germany). Imaging comprised: a coronal T2-weighted turbo spin echo (TSE) sequence, a sagittal T1-weighted magnetization prepared rapid gradient echo (MPRAGE) sequence with submillimeter spatial resolution, an axial susceptibility weighted sequence (SWI), a T1-weighted true inversion recovery (tIR) sequence and a diffusion weighted (DWI) sequence. MR images were assessed for image quality and scored for pathology to assign study subjects into two groups 1) patients without postoperative structural white matter injury and 2) patients with postoperative structural white matter injury assessed as perioperatively acquired. Reviewing and scoring of MRI were done by two pediatric neuroradiologists blinded to biomarker data. Consensus data are presented.

### 2.5. Statistics

Analyses were performed using absolute biomarker concentration data for pre- and postoperative analyses, and adjusted biomarker data at the respective timepoints for postoperative analyses. For analyses using absolute brain injury marker concentrations, biomarker concentrations were $\log_{10}$-transformed prior to analysis due to the non-parametric distribution of raw data. Adjusted biomarker data were defined as (postoperative concentrations − preoperative concentrations) ÷ preoperative concentrations expressed as percentage change from baseline.

Comparisons between timepoints were done using repeated measures ANOVA with post hoc Bonferroni correction. Adjusted p-values≤0.05 were considered significant. The relationship between absolute and adjusted postoperative biomarker concentrations and presence of postoperative structural white matter injury was evaluated in a subset of study participants with assessable postoperative brain MRI (n=30). Wilcoxon rank sum test was used for comparisons between the group with postoperative white matter injury and the group without postoperative white matter injury. Results were corrected for multiple comparisons using Bonferroni correction. Adjusted p-values≤0.05 were considered significant.

Sex, birthweight, postnatal age at surgery (days), highest preoperative lactate (mmol/L), preoperative balloon atrial septostomy (y/n) and presence of preoperative white matter lesion (y/n) were assessed for possible associations with preoperative biomarker absolute concentrations using univariable and multivariable linear regression models. Preoperative white matter lesion was a rare event in the study cohort (1/24) and thus excluded from analysis. RACHS-1 score, corrective or palliative procedure, time on bypass circulation (minutes), selective cerebral perfusion (y/n), ultrafiltration when on bypass (y/n) and highest postoperative lactate (mmol/L) were assessed for possible correlations with postoperative day 2 absolute concentrations with univariable and multivariable linear regression models. Postoperative day 2 samples were chosen recognizing the significant increase in GFAP and Tau at postoperative day 2 compared to preoperative concentrations, and to obtain the maximal number of data points. No adjustment for multiple comparisons were made due to the exploratory nature of the study. Unadjusted p-values≤0.05 were considered significant.

All statistical analyses were performed using R version 4.2.0 (15).

## 3. Results

### 3.1. Perioperative plasma brain injury marker concentrations

Preoperative, and postoperative day 1 and 2 blood samples were obtained in 40 subjects. Postoperative day 3 samples were obtained in 27 participants. Plasma concentrations of all measured brain injury markers in all acquired samples were above the lower limit of detection.

**Figure 1:**
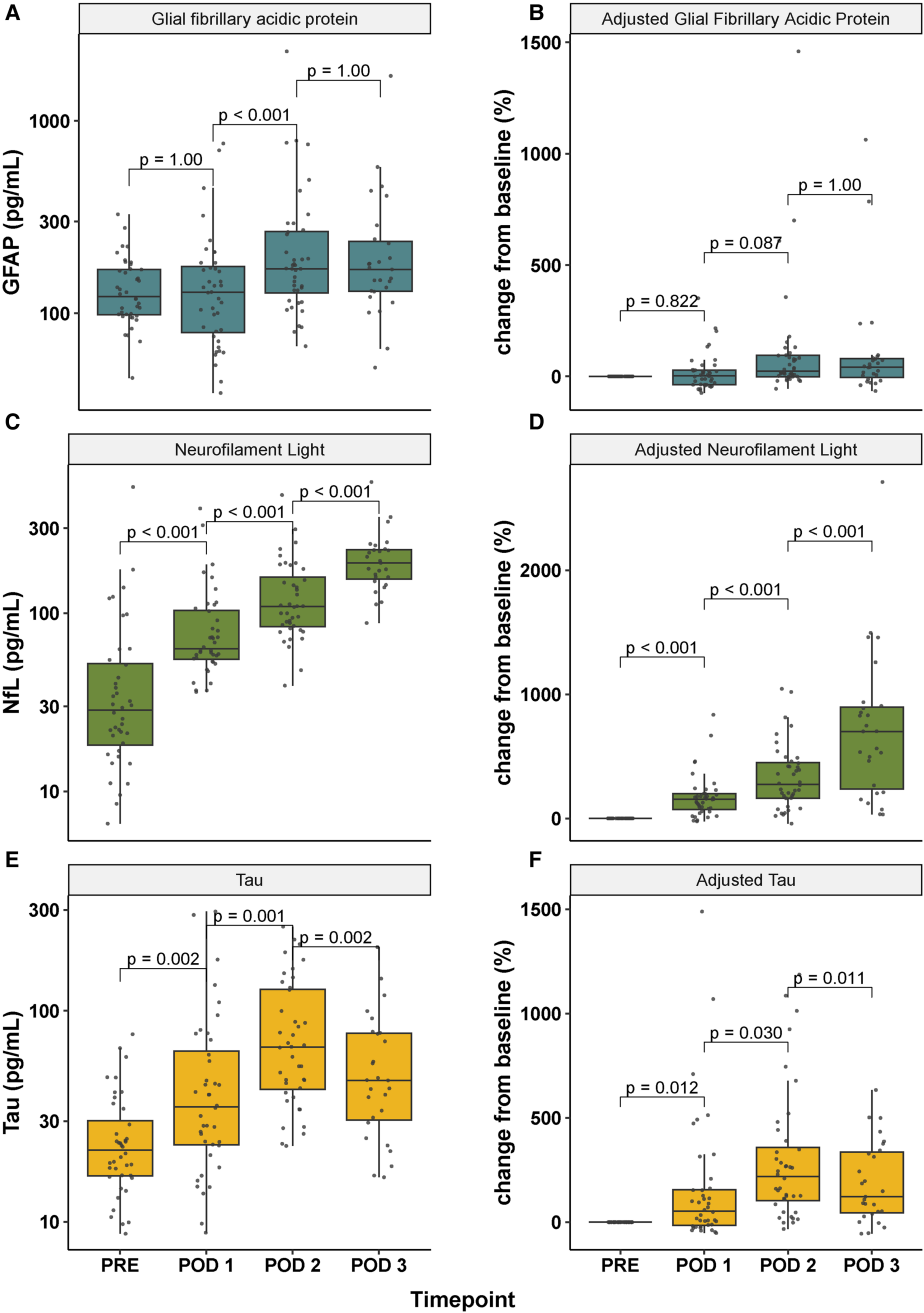
Temporal trajectory of absolute (**A**, **C**, **E**) and adjusted (**B**, **D**, **F**) plasma concentrations of glial fibrillary acidic protein (GFAP), neurofilament light (NfL) and Tau before and after neonatal open-heart surgery. Note that the y-axis in panel A, C and E is presented on a $\log_{10}$-scale. Baseline in panel B, D and F is set to preoperative concentrations. GFAP, glial fibrillary acidic protein; NfL, neurofilament light; PRE, preoperative; POD 1–3, postoperative day 1–3.

Postoperative day 1–3 plasma brain injury marker concentrations displayed a temporally well-defined trajectory. GFAP median concentrations increased significantly at postoperative day 2 compared to preoperative and postoperative day 1 concentrations, and remained at comparable levels at postoperative day 3. Postoperative concentrations of GFAP adjusted for preoperative concentrations were not significantly different between timepoints, Figures 1A,B. Median absolute concentrations of NfL increased twofold from preoperative concentrations at postoperative day 1, and continued to rise each postoperative day corresponding to a seven-fold increase at postoperative day 3, Figures 1C,D. Median concentrations of Tau reached peak concentrations at postoperative day 2, equaling a three-fold increase compared to preoperative concentrations. Tau concentrations at postoperative day 3 were significantly decreased in comparison with postoperative day 2 concentrations, Figures 1E,F. Absolute and adjusted plasma brain injury marker concentrations and distributions are presented in Table 2.

**Table 2:** Absolute and adjusted plasma concentrations for brain injury markers Glial fibrillary acidic protein, Neurofilament Light and Tau.

Brain injury marker	Concentration (pg/ml)	Change from preoperative concentrations (%)
	(median (IQR))	(median (IQR))
Glial fibrillary acidic protein		
preoperative	122.3 (98.2–169.0)	
postoperative day 1	128.4 (79.2–175.0)	3 (−37–28)
postoperative day 2	170.5 (127.7–266.0)	24 (−1–94)
postoperative day 3	169.0 (130.2–236.7)	42 (−4–80)
Neurofilament Light		
preoperative	28.6 (18.2–52.2)	
postoperative day 1	63.1 (55.1–103.6)	154 (73–199)
postoperative day 2	108.9 (84.2–159.3)	275 (164–449)
postoperative day 3	191.5 (155.2–227.4)	701 (238–897)
Tau		
preoperative	21.9 (16.5–30.3)	
postoperative day 1	35.1 (23.2–64.6)	54 (−15–156)
postoperative day 2	67.2 (42.5–126.3)	219 (103–359)
postoperative day 3	46.8 (30.3–78.1)	122 (45–336)

### 3.2. Magnetic resonance imaging

Preoperative and postoperative MRI scans were obtained in 24 and 33 study subjects, respectively. Three postoperative scans were excluded due to insufficient quality generating a total of 54 scored examinations. Pre- and postoperative imaging were available in 20 study participants. Four subjects had preoperative imaging only, and ten study participants had postoperative imaging only. The preoperative MRI examination was obtained at a median of 1 day (IQR 1–3) before surgery. The postoperative MRI examination was performed at a median of 8 days (IQR 6–10) post surgery.

Postoperative MRI did not show any structural white matter lesion in 14 study participants. White matter injury was detected in 16 subjects, Table 1. One of these subjects presented with a single punctate lesion in the preoperative MRI. The same subject had a substantial aggravation of white matter lesions in the postoperative MRI and was thus included in the subgroup with postoperative white matter injury in subsequent analysis. In study participants with postoperative white matter injury and lacking preoperative MRI (n=7), white matter lesions were scored as perioperatively acquired if not assessed as related to birth trauma or preexisting comorbidity. Identified white matter injury comprised multiple punctate lesions in 14 subjects with restricted diffusion in six study participants and SWI-verified hemorrhage in one subject, and SWI-verified white matter microbleeds in two study subjects.

### 3.3. Predictive ability of brain injury marker concentrations for postoperative structural white matter injury

**Figure 2:**
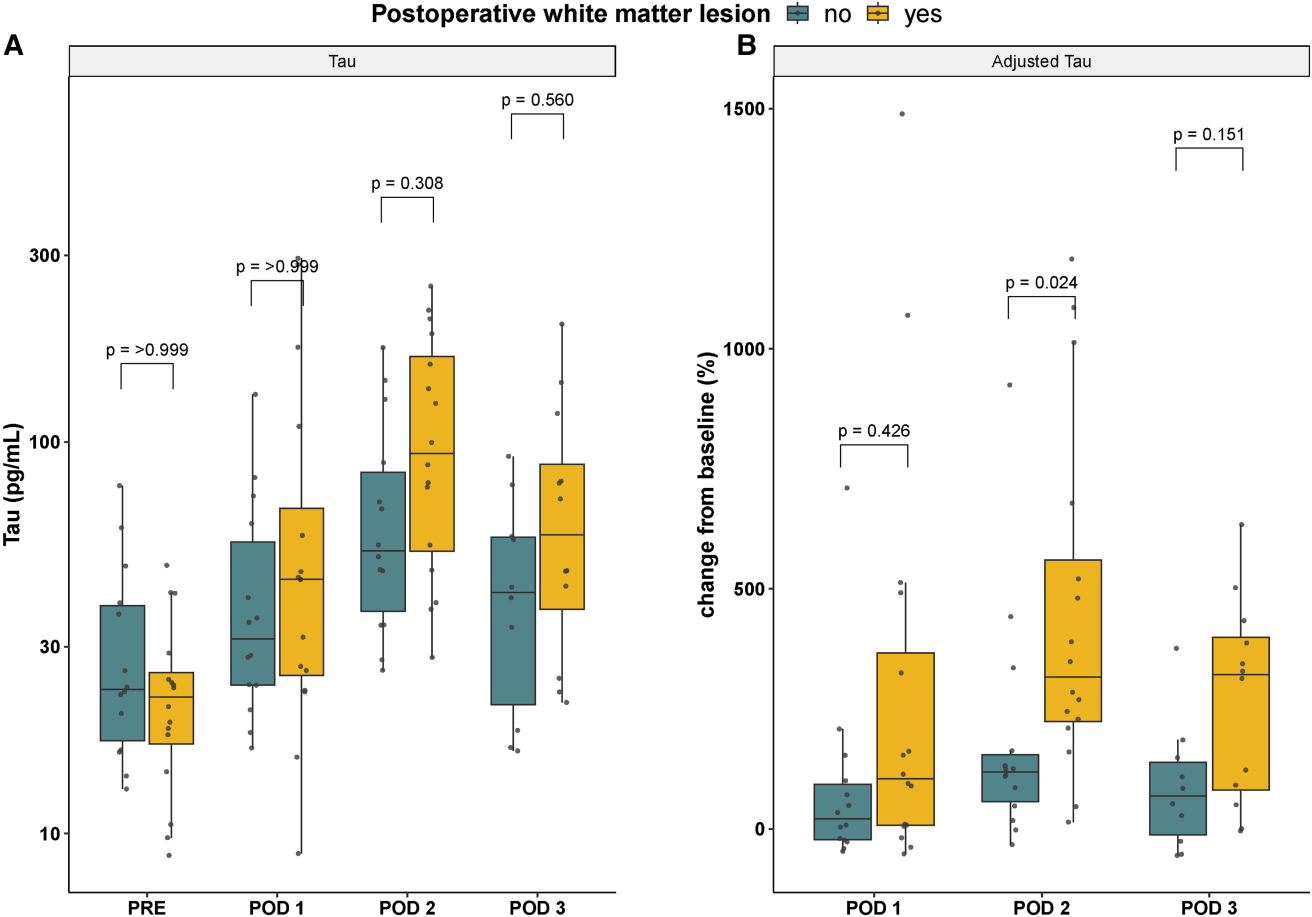
Absolute (**A**) and adjusted (**B**) Tau concentrations stratified according to presence (yellow) or absence (blue) of white matter injury at the postoperative brain MRI scan. Note that the y-axis in panel A is presented on a $\log_{10}$-scale. Baseline is set to preoperative concentrations in panel B. GFAP, glial fibrillary acidic protein; NfL, neurofilament light; PRE, preoperative; POD 1–3, postoperative day 1–3.

The relative increase in plasma Tau from preoperative concentrations until postoperative day 2 was significantly higher in infants presenting with white matter injury at the postoperative brain MRI compared to that in infants without white matter lesions. No statistically significant differences in the absolute concentrations of Tau were observed when stratified for presence or absence of postoperative white matter injury, Figures 2A,B. Postoperative day 2 concentrations of Tau were significantly increased in infants undergoing a palliative procedure as compared to neonates subjected to a corrective procedure, see below. A *posthoc* subgroup analysis restricted to neonates subjected to a corrective procedure (n=22) was made, demonstrated a persisting significant correlation between increased adjusted Tau at postoperative day 2 and subsequent white matter injury, p=0.04.

Absolute or adjusted plasma concentrations of GFAP and NfL respectively were not associated with postoperative white matter injury at any timepoint, data not shown.

### 3.4. Associations between clinical variables and perioperative biomarker concentrations

**Figure 3:**
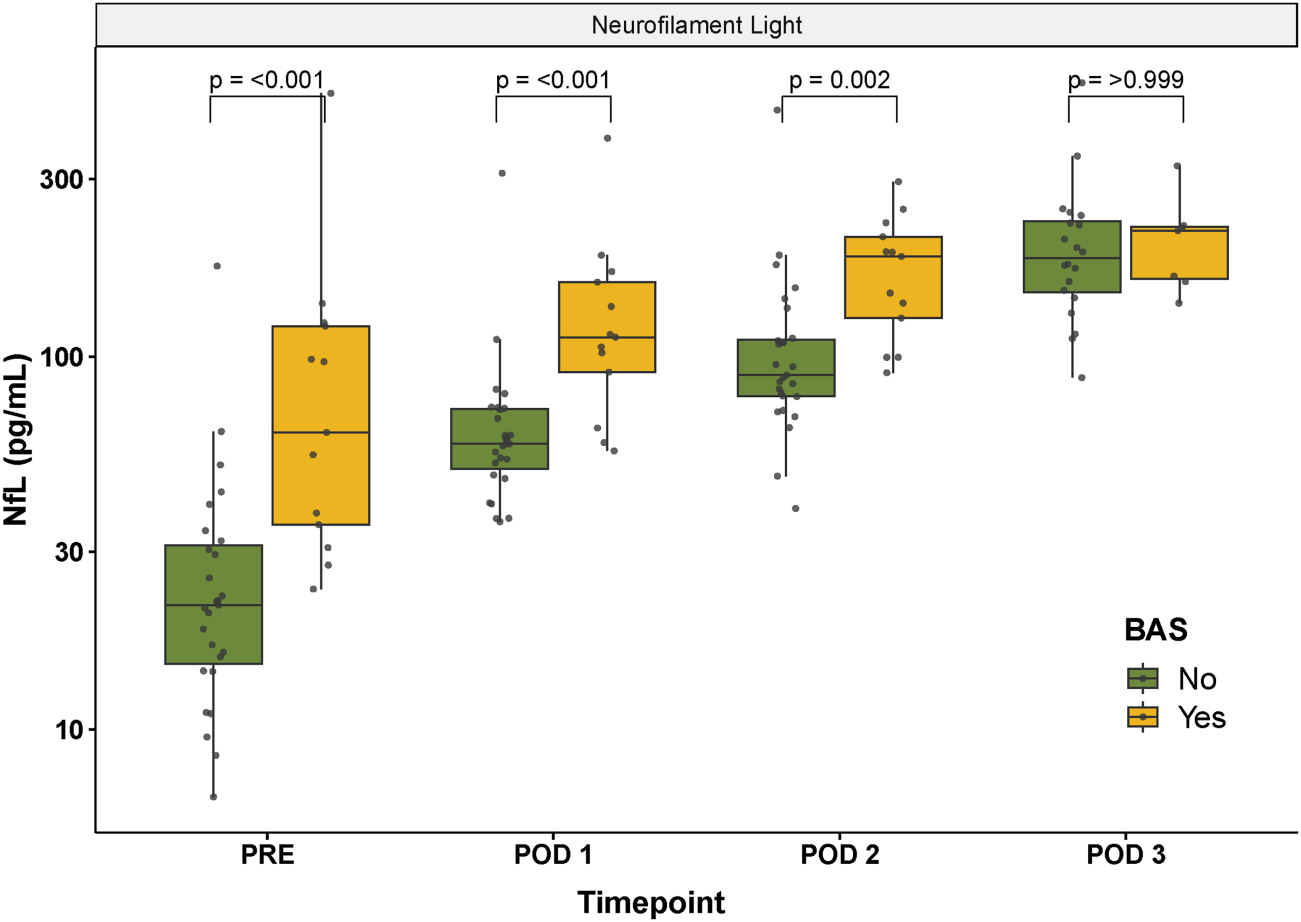
Perioperative NfL concentrations stratified according to previous balloon atrial septostomy yes/no. Note that the y-axis is presented on a $\log_{10}$-scale. NfL, neurofilament light; PRE, preoperative; POD 1–3, postoperative day 1–3; BAS, balloon atrial septostomy.

Preoperative NfL concentrations were increased in infants subjected to balloon atrial septostomy (BAS), Figure 3. The association between preoperative BAS and increased plasma concentrations of NfL remained significant at postoperative day 1 and 2, but not at postoperative day 3, Figure 3. The relationship between previous BAS and increased perioperative concentrations of NfL persisted after adjustment for pre- and postoperative maximal lactate concentrations and RACHS-1 group, respectively. Neonates undergoing a palliative procedure presented with increased Tau median concentrations at postoperative day 2 compared to neonates undergoing corrective surgery (127 pg/ml for palliative procedure vs 55 pg/ml for corrective surgery, p=0.01). Beta estimates and confidence intervals for significant correlations at postoperative day 2 are presented in Table 3.

**Table 3:** Univariable and multivariable regression analysis of postoperative day 2 brain injury marker concentrations. Bold face indicate statistical significance defined as p≤0.05.

Variable	Univariable model		Multivariable model	
	β	[95% CI]	β	[95% CI]
**Neurofilament light**				
BAS = yes	**0.22**	**[0.09, 0.35]**		
max lactate (mmol/L)	**0.08**	**[0.007 , 0.14]**	0.04	[−0.03 , 0.11]
+ BAS = yes			**0.19**	**[0.05 , 0.32]**
RACHS-1 = 4	− **0.17**	**[−0.30, −0.03]**	−0.06	[−0.19 , 0.08]
+ BAS = yes			**0.23**	**[0.09 , 0.37]**
**Tau**				
corr proc = yes	**−0.28**	**[−0.48 , −0.07]**		

BAS, balloon atrial septostomy; RACHS-1, risk adjustment for congenital heart surgery.

Sex, birthweight and postnatal age at surgery were not associated with preoperative plasma brain injury marker concentrations as determined by univariable linear regression analysis. Brain injury marker concentrations at postoperative day 2 were not correlated to selective cerebral perfusion or ultrafiltration.

## 4. Discussion

This study reports on the perioperative trajectory of plasma brain injury markers GFAP, NfL and Tau, and their respective association with postoperative white matter lesions following neonatal open-heart surgery on cardiopulmonary bypass circulation. We observed a characteristic response over time for the respective brain injury markers from preoperative concentrations through the first postoperative days. Neonates presenting with postoperative structural white matter injury had a more pronounced relative increase in Tau at postoperative day 2 compared to infants without postoperative white matter injury. The difference in absolute plasma Tau concentrations between the groups was however not significant. Neither GFAP nor NfL were able to discriminate between infants with or without white matter injury at any timepoint.

The finding of a pronounced relative increase in Tau concentrations in infants with postoperative white matter lesions deserves some consideration. First, the high prevalence of postoperative white matter lesions in our study supports numerous previous reports on the vulnerability of the immature white matter in the neonate born with a critical congenital heart defect (2,16). The majority of the postoperative white matter lesions detected by MRI in our cohort were not identifiable by any clinically overt neurological symptomatology, advocating the call for a prognostic biomarker. Second, our finding of increased adjusted Tau in children with subsequent white matter injury is in line with previous studies evaluating Tau in pediatric brain injury from other causes, e.g. traumatic brain injury, cerebral malaria and anoxic brain injury (17–19). In those studies, increased circulating concentrations of Tau have been associated with worse clinical presentation or adverse neurocognitive outcome. Third, the dichotomization of postoperative white matter injury into a yes/no-variable prevented a more granular analysis of the association between adjusted Tau concentrations and postoperative white matter injury.

It is debatable whether the evaluation of associations between intraoperative events and postoperative brain injury marker concentrations requires adjustment for the respective preoperative concentrations. Neonatal open-heart surgery on cardiopulmonary bypass circulation results in an extensive dilution of the native circulating blood volume which hypothetically minimizes the effect of preoperative protein concentrations on the postoperative concentrations. Although the circuit volume per bodyweight was similar between subjects in this study, the fresh frozen plasma transfusion requirements after bypass separation were significantly different between study subjects (0–116 ml/kg). Moreover, it is plausible that uncorrected critical congenital heart defects with aberrant hemodynamics have a significant effect on preoperative plasma brain injury marker concentrations. This was clearly demonstrated by the correlation between previously performed BAS and increased circulating concentrations of NfL. While acknowledging these limitations, we chose to present adjusted and absolute concentrations in this study recognizing the added information of adjusted values.

Previous studies reporting on GFAP concentrations in infants subjected to neonatal cardiac surgery have shown significant increases in biomarker concentrations immediately after bypass separation (6,8). None of the cited studies evaluated GFAP concentrations beyond the day of surgery, making comparisons difficult. When contrasting our biomarker concentrations with results obtained at comparable timepoints in adult cardiac surgery (20,21), maximal NfL and Tau concentrations were ten-fold higher in neonates compared to those observed in adults. One of the studies identified a substantial increase in plasma Tau concentrations two hours after cardiopulmonary bypass circulation (21). Evaluation of plasma biomarker concentrations at bypass separation or at PICU admission was unfortunately lacking in our material but should be included in upcoming studies.

Our study is limited by the small cohort, the single-centre design and the lack of a control group. Moreover, assessment of the prognostic ability of a biomarker for subsequent white matter injury would preferably incorporate objective measures of white matter function and maturation, e.g., diffusion tensor imaging data enabling a sensitive evaluation of white matter microstructure (22,23). The lack of quantified white matter integrity apart from structural injury is a limitation of the present study, as is the lack of long term neurodevelopmental outcome data.

We present novel perioperative data on GFAP, NfL and Tau plasma concentrations in neonates subjected to open heart surgery and propose that a pronounced increase in Tau at postoperative day 2 may serve as a prognostic biomarker for postoperative white matter injury. These data will be of value in the design of larger trials with the objective of identifying and validating the prognostic ability of circulating brain injury markers for subsequent white matter injury and neurodevelopmental impairment.

## Data Availability

The datasets presented in this article are not readily available because of patient confidentiality, but can be made available upon reasonable request. Requests to access the datasets should be directed to asa.jungner@med.lu.se.
